# Molecular Insights into Elevated Autoantibodies in Polycystic Ovary Syndrome: Mechanisms and Clinical Implications

**DOI:** 10.3390/ijms26178192

**Published:** 2025-08-23

**Authors:** Jakub Kwiatkowski, Nicole Akpang, Zofia Ziemkiewicz, Lucja Zaborowska, Artur Ludwin

**Affiliations:** 1 1st Department of Obstetrics and Gynecology, Medical University of Warsaw, 02-015 Warsaw, Poland; s084954@student.wum.edu.pl (N.A.); zaborowska.lucja@doctoral.uj.edu.pl (L.Z.); 2 Department of Biochemistry, University of Oxford, Oxford OX1 3QU, UK; zofia.ziemkiewicz@oriel.ox.ac.uk; 3 Doctoral School of Medical and Health Sciences, Jagiellonian University Collegium Medicum, 31-530 Cracow, Poland

**Keywords:** polycystic ovary syndrome, PCOS, autoimmunity, autoimmune diseases, autoantibodies, inflammation

## Abstract

Polycystic ovary syndrome (PCOS) is a prevalent endocrinological condition among women of reproductive age, characterized by several well-known symptoms, including hyperandrogenism, anovulation, irregular menstrual cycles, and insulin resistance. In addition, women suffering from PCOS are also at an increased risk of developing several autoimmune diseases, including thyroid disorders, type 1 diabetes, and rheumatoid arthritis. Furthermore, an elevated prevalence of diverse autoantibodies is observed in women diagnosed with PCOS. These include antibodies specific to autoimmune diseases, e.g., anti-thyroid peroxidase (anti-TPO), anti-thyroglobulin (anti-TG), and antinuclear antibodies (ANAs), as well as those that are non-specific, such as anti-malondialdehyde-modified human serum albumin (anti-HSA-MDA) or anti-α-crystallin. It appears that several mechanisms may be responsible for this phenomenon. PCOS has been observed to co-occur with autoimmune diseases, potentially attributable to shared genetic susceptibility or the presence of hormonal disorders resulting from autoimmune diseases. Moreover, PCOS is a chronic low-grade inflammatory disease that may contribute to immune dysfunction and subsequent overproduction of autoantibodies. A further intriguing aspect may be the yet-unknown role of autoantibodies in the pathogenesis of PCOS, considering PCOS as a disease with an autoimmune etiology.

## 1. Introduction

Approximately 1 in 15 premenopausal women worldwide are affected by polycystic ovary syndrome (PCOS), an endocrine and metabolic disorder associated with hyperandrogenism and ovarian dysfunction [1]. Although PCOS is the most common endocrine disorder among women of reproductive age, a significant proportion of affected individuals remain undiagnosed. In the American population, this accounts for up to 75% of women with PCOS [2]. Although PCOS was first described in 1935 by Stein and Leventhal, and since then, up to 30 genes have been linked to the development of this disease, its pathophysiology remains not fully elucidated [2]. Furthermore, the criteria for its diagnosis remain a topic of ongoing discussion [1]. The difficulties in establishing clear diagnostic criteria arise from the heterogeneity of the disease and the variety of symptoms experienced by patients. Diagnosis remains challenging, as it requires the identification of specific characteristics of the syndrome while also excluding other potential causes of these symptoms such as thyroid disease, hyperprolactinemia, androgen-secreting tumors, and nonclassical congenital adrenal hyperplasia [3]. Currently, the most widely used and accepted criteria are the Rotterdam criteria, which state that the diagnosis requires the presence of at least two of the following three features: (1) biochemical or clinical hyperandrogenism; (2) oligo- or anovulation; (3) polycystic ovarian morphology (PCOM) [4,5]. Serum anti-Müllerian hormone (AMH) has been suggested as an alternative marker for PCOM in adults, thereby potentially supporting the diagnosis of PCOS. AMH could help define PCOM in adults, but it should not be used in the adolescent population or as the sole indicator of PCOS [5]. PCOS is the leading cause of anovulatory infertility, being responsible for approximately 80% of such cases [6,7]. Beyond infertility, affected women experience various clinical manifestations, including hirsutism, acne, obesity, insulin resistance, and menstrual irregularities [4]. The consequences of PCOS extend beyond these symptoms, resulting in a significantly higher risk of type 2 diabetes, endometrial cancer, underlying cardiovascular disease, mood disorders, and depression [8,9,10,11]. Additionally, PCOS is associated with an increased risk of obstetric complications, such as spontaneous abortion, preeclampsia, pregnancy-induced hypertension, and gestational diabetes [12,13,14]. In addition to the higher prevalence of the aforementioned conditions in patients with PCOS, autoimmune diseases, including thyroid disorders, type 1 diabetes, rheumatoid arthritis, and Sjögren’s syndrome, are also more commonly observed [15,16,17]. This association often works bilaterally: up to 25% of patients with type 1 diabetes will develop PCOS [18]. Furthermore, patients with PCOS exhibit elevated levels of various autoantibodies compared to healthy individuals [19,20,21,22]. Significant research has been conducted over the years to explore why these autoantibodies are more prevalent and what their implications or underlying causes might be. Could immune system dysfunction, triggered by chronic systemic inflammation, be the underlying cause?

## 2. PCOS, Autoimmune Diseases, and Related Autoantibodies—Current Evidence

There is a clear association between PCOS and an increased prevalence of autoimmune diseases, including both organ-specific autoimmunity (e.g., Hashimoto’s thyroiditis) and systemic autoimmunity (e.g., systemic sclerosis) [23] (Table 1). Additionally, various autoantibodies correlated with these conditions have been investigated in women with PCOS, which will be discussed in detail below.

### 2.1. Autoimmune Thyroiditis

Thyroid disorders are an exclusion criterion for the diagnosis of PCOS [2,15], meaning that the prevalence of clinical thyroid dysfunction should theoretically be zero in the PCOS population. However, many studies report the prevalence of autoimmune thyroiditis (AIT) in patients with PCOS, raising questions about the overlap between these conditions. Exploring the frequency of thyroid disorders in PCOS cohorts and the potential reasons for its reported presence could provide valuable insights into the diagnostic criteria and underlying pathophysiology [15].

One of the most extensively studied connections among autoimmune diseases is the link between AIT (Hashimoto’s disease) and PCOS. This relationship has been confirmed in meta-analyses, which have demonstrated a higher prevalence of Hashimoto’s disease among women with PCOS. In a 2022 systematic review and meta-analysis, which included 18 studies, PCOS patients were shown to have a higher risk of developing Hashimoto’s thyroiditis, with an odds ratio (OR) = 2.28 (95% confidence interval (95% CI): 1.61–3.22) compared to non-PCOS women [25]. Similarly, an analysis of three studies reported an OR of 4.81 (95% CI: 2.88–8.04) [26]. Another meta-analysis of 18 studies found a comparable risk (OR = 2.38, 95% CI: 1.63–3.49) [27]. The explanation for this connection is sought at the genetic and immunological levels, as well as in the numerous hormonal disturbances shared between PCOS and Hashimoto’s disease.

Both PCOS and Hashimoto’s thyroiditis show strong genetic influences on inheritance and disease susceptibility. While only a few genetic variants have been linked to the co-occurrence of these conditions, three key polymorphisms have been identified: fibrillin 3 (*FBN3*) intronic D19S884 allele 8 (A8), possibly affecting its transcript splicing and associated with lower transforming growth factor β (TGF-β) activity and regulatory T cell (Treg) levels; *CYP1B1* L432V polymorphism (rs1056836), involved in estrogen metabolism and associated with serum T4, fT4, and fT3 concentrations in PCOS patients; and gonadotropin-releasing hormone receptor (*GnRHR*) 3′-UTR polymorphism (rs1038426), affecting its expression, related to gonadotropin-releasing hormone signaling, concentration of serum TSH, and insulin signaling in women diagnosed with PCOS [33]. Additionally, genome-wide association studies (GWASs) have pinpointed two shared susceptibility loci: follicle-stimulating hormone receptor (*FSHR*) and insulin receptor (*INSR*) genes [34]. However, no functional connection between the two disorders has been established.

Thyroid antibodies have been detected in ovarian follicular fluid, showing a correlation with their serum levels [35]. A potential mechanism suggested that anti-thyroid peroxidase (anti-TPO) antibodies may pass through the blood–follicle barrier during follicular development, leading to the destruction of and damage to growing follicles and oocytes. This indicates that thyroid antibodies may have a direct impact on ovarian tissue. Furthermore, since the zona pellucida and thyroid tissue appear to share similar antigens, the zona pellucida may also become a target for anti-thyroid antibodies [35,36].

Higher serum estradiol levels were observed in anti-TPO antibody-positive women with PCOS compared to those who were anti-TPO antibody-negative, with a positive correlation found between anti-TPO antibody levels, estradiol, and the estradiol/progesterone ratio [37]. These results were recently confirmed in adolescent females with PCOS [38], which may support the hypothesis that elevated estrogen levels, in combination with reduced progesterone, contribute to excessive immune system activation.

The immunosuppressive effects of androgens make the role of hyperandrogenism in AIT development in PCOS unclear, and this hypothesis remains difficult to assess based on the available literature [15]. Patients with both PCOS and AIT exhibited lower testosterone levels, free androgen index, and hyperandrogenemia compared to those with PCOS alone [39]. While AIT-related menstrual cycle disturbances could potentially affect androgen levels, no significant differences in cycle abnormalities or ovarian cyst prevalence were found between groups. These findings align with the hypothesis that hormonal disturbances in PCOS may contribute to autoimmune processes while also reflecting the immunosuppressive effects of androgens. In contrast, another study reported a higher prevalence of thyroid disorders, including AIT, in women with the full PCOS phenotype, characterized by PCOM, oligo- or anovulation, and hyperandrogenism [40].

Beyond the increased prevalence of Hashimoto’s thyroiditis in PCOS, available research has also investigated the presence of its characteristic autoantibodies (anti-TPO and anti-thyroglobulin (anti-TG)) in euthyroid women with PCOS or those without a diagnosis of thyroid disease. The results are inconsistent; some indicate a higher prevalence of thyroid autoantibody positivity in euthyroid PCOS patients or significantly elevated thyroid autoantibody titers in women with PCOS compared to controls. For instance, it was demonstrated that euthyroid women with PCOS had a 4.88-fold higher prevalence of anti-TPO antibody positivity (95% CI: 2.40–9.95) and a 3.39-fold higher prevalence of anti-TG antibody positivity (95% CI: 1.24–9.28) compared to the control group [20]. Similarly, a prospective multicenter study reported elevated anti-TPO or anti-TG antibodies in 14 out of 168 controls (8.3%) and in 47 out of 175 patients with PCOS (26.9%; *p* < 0.001) [41]. On the other hand, other researchers did not find statistically significant differences in the frequency of serum anti-TPO antibody positivity between women with PCOS and controls [42,43]. The interpretation and synthesis of findings across studies are highly challenging due to the heterogeneity of study designs, varying patient inclusion criteria, and even differences in the diagnostic criteria for PCOS. A recent meta-analysis of 40 observational studies found a significantly higher prevalence of thyroid autoantibodies in PCOS: anti-TPO OR = 2.03 (95% CI, 1.35–3.04; *p* = 0.0006) and anti-TG OR = 1.92 (95% CI, 1.23–3.01; *p* = 0.004) [44].

In summary, the predisposition to the simultaneous occurrence of PCOS and Hashimoto’s thyroiditis is best explained by a multidirectional interplay of factors. A central element in this connection is immune system dysfunction, particularly the dysregulation of Treg cells, which links genetic and functional networks between the two disorders [33,45,46]. Additionally, the unique hormonal and metabolic profile observed in women with PCOS may create conditions that promote the development of thyroid autoimmunity [33].

### 2.2. Graves’ Disease

Data regarding other thyroid disorders is very sparse [15]. Regarding Graves’ disease (GD), only a few publications have been released, including one large cohort study, which indicates that the adjusted hazard ratio for PCOS in patients with GD compared to those without GD was 1.47 (95% CI: 1.09–1.98), suggesting that women with GD could be at risk of developing PCOS [28]. However, supporting data for such an observation is very limited, and there is a lack of data on autoantibodies in this context.

### 2.3. Type 1 Diabetes Mellitus

Among autoimmune diseases, particular attention has been given to the coexistence and interplay between type 1 diabetes (T1D) and PCOS. According to a meta-analysis of 13 studies, the pooled prevalence of PCOS in women with T1D, based on the Rotterdam 2003 criteria, was as high as 26% [18]. Insulin resistance is central to the pathogenesis of PCOS, whereas β-cell dysfunction and insulin deficiency are key factors in T1D development [18]. Although obesity, insulin resistance, and endogenous hyperinsulinemia are not prerequisites for T1D, its treatment involves exogenous subcutaneous insulin administration, leading to supraphysiological insulin concentrations in the systemic circulation [47]. This directly affects peripheral tissues, including ovarian theca cells, stimulating ovarian steroidogenesis [48]. In women with T1D and PCOS, sex hormone-binding globulin (SHBG) concentrations remain comparable to those of non-PCOS women with T1D or healthy controls, limiting free testosterone availability despite increased total testosterone. This may explain the milder hyperandrogenic phenotype in these patients and contribute to the underdiagnosis of PCOS in T1D [24,47].

Beyond the well-established role of insulin, epigenetic mechanisms have also been implicated in the coexistence of T1D and PCOS. Epigenome-wide association studies (EWASs) have identified immune- and autoimmunity-related pathways altered in PCOS, notably the T1D pathway, antigen processing and presentation, and interferon signaling [49,50,51]. In Kyoto Encyclopedia of Genes and Genomes (KEGG) analyses integrating differentially methylated and differentially expressed genes, the T1D pathway consistently ranks among the top enriched terms [51,52]. In ovarian tissue, integrative methylome–transcriptome profiling revealed coordinated methylation–expression changes in *HLA-B* and *HLA-F*, indicating that the enrichment of the T1D pathway is largely driven by signals from the major histocompatibility complex (MHC) region and supporting the involvement of MHC-mediated antigen presentation in crosstalk between PCOS and autoimmunity [52]. Consistently, whole-blood EWASs in PCOS also show enrichment for the T1D pathway and a prominent human leukocyte antigen (HLA) region signal, suggesting convergent immuno-epigenetic mechanisms relevant to T1D comorbidity [51]. However, there is a lack of studies investigating the presence of T1D-specific autoantibodies in women with PCOS. One study addressing this issue evaluated the prevalence of anti-glutamic acid decarboxylase 65 (anti-GAD65) and anti-insulinoma-associated antigen-2 (anti-IA2) autoantibodies in pregnant women with gestational diabetes mellitus (GDM), normal pregnancy, and PCOS to assess whether GDM in PCOS is partially induced by autoantibodies. However, their frequency was very low in both normal pregnancy and PCOS groups [53].

### 2.4. Antinuclear Antibody-Related Diseases

The role of antinuclear antibodies (ANA) as serologic parameters of autoimmunity is frequently emphasized. Many rheumatic diseases are characterized by the presence of one or more of these ANAs, such as systemic lupus erythematosus (SLE), systemic sclerosis, Sjogren’s syndrome, mixed connective tissue disease (MCTD), polymyositis, dermatomyositis, and rheumatoid arthritis (RA) [54,55]. The association between PCOS and rheumatic diseases is also reflected in research analyzing their prevalence. In a large prospective cohort study, a history of polycystic ovary syndrome was found to be positively associated with RA (relative risk (RR) = 2.58; 95% CI: 1.06–6.30). Moreover, in multivariate analysis models, a history of PCOS remained the most consistent predictor of RA [29]. Similarly, an increased prevalence of Sjögren’s syndrome [30], systemic sclerosis, and undifferentiated connective tissue disease has been reported among women with PCOS [16].

Several studies have focused on analyzing the presence of these antibodies in women with PCOS, particularly highlighting anti-double-stranded DNA (anti-dsDNA) and anti-histone antibodies [22,56,57,58]. While the results are not unanimous, most studies suggest higher levels of ANAs or more frequent ANA positivity in women with PCOS. In a prospective case–control study, it was shown that women with PCOS had significantly elevated serum levels of anti-histone and anti-dsDNA antibodies [56]. Similarly, Rashid et al. reported a statistically significant difference (*p* < 0.01) in ANA positivity between PCOS patients (18.4%) and the control group (2.29%) [22]. It has also been hypothesized that laparoscopic ovarian electrocauterization has the potential to trigger increased autoimmune responses in PCOS patients. A significant rise in the number of ANA-positive cases was observed following the procedure, increasing from 3 out of 35 (8.6%) before electrocauterization to 10 out of 35 (28.6%) after, while none of the control subjects tested positive [57]. The predominant ANA subtype identified in positive samples was anti-SSA (anti-Sjögren’s-syndrome-related antigen A). These findings suggest that laparoscopic ovarian electrocauterization may expose ovarian antigens to the immune system, thereby eliciting autoimmune reactions in affected patients. However, some studies do not confirm these observations. For instance, according to Hamedi et al., no significant differences were found in ANA levels between PCOS patients and the control group [58]. Nevertheless, the majority of studies suggesting such an association conclude that the high prevalence of ANA positivity among women with PCOS may serve as an indicator of autoimmunity as a potential underlying cause of the syndrome [22,56].

### 2.5. Beyond Organ-Specific Autoimmunity

It is also important to mention the numerous other autoantibodies that have been investigated in women with PCOS. However, these are not necessarily associated with well-characterized autoimmune diseases, and when they are, the available study results remain very limited and heterogeneous. The presence of common autoantibodies, including nuclear (ANAs), smooth muscle (SMAs), parietal cell (PCAs), thyroid microsomal (TMAs), reticulin (ARAs), mitochondrial (AMAs), and liver/kidney microsomal antibodies (LKMAs), as well as anti-β2-glycoprotein I (anti-β2GPI) and anti-carbonic anhydrase (anti-CA), was investigated in women with reproductive failure. Among these, ANAs and SMAs were found to be more prevalent in women with PCOS compared to controls [59]. However, the study was conducted on a small sample size (20 patients with PCOS), which may have limited its statistical power to detect differences in the prevalence of the other autoantibodies. Similarly, a 2006 study investigated the presence of common autoantibodies, including ANAs, SMAs, PCAs, TMAs, AMAs, anti-β2GPI, and cardiolipin antibodies, in infertile women undergoing in vitro fertilization (IVF). PCOS was associated with a higher number of positive autoantibody tests compared to tubal factor infertility, with 5/21 (23.8%; 95% CI: 9.11–47.5) vs. 6/56 (10.7%; 95% CI: 4.4–22.6), respectively [60]. Interestingly, it was demonstrated that infertile patients with PCOS exhibit significantly higher levels of anti-endometrial antibodies (AEAs), antibodies against malondialdehyde-modified human serum albumin (anti-HSA-MDAs), and oxidized proteins (protein–MDA) compared to age- and BMI-matched controls. These findings support the hypothesis that oxidative stress and autoimmune responses may contribute to an abnormal endometrial environment, leading to poor embryo receptivity in infertile PCOS patients. The generation of AEAs in PCOS could reflect an immune response to newly oxidized protein epitopes formed during oxidative stress in endometrial cells [61]. Moreover, elevated concentrations of anti-α-crystallin antibodies were observed in patients with PCOS. Alpha-crystallin, a major water-soluble lens protein, belongs to the heat shock protein (HSP) family. These proteins have long been recognized as antigens with high organ specificity and low species specificity, remaining in immunological isolation and serving as potential autoantigens. The findings suggest that the increased production of anti-α-crystallin antibodies in women with PCOS is most likely due to a failure of immune tolerance, leading to an induced immune response. This is presumably driven by the heightened expression of this stress protein in response to oxidative stress and chronic inflammation [19].

A summary of the results of key studies on autoantibodies in women with PCOS is presented in Table 2.

### 2.6. Autoantibodies Targeting Hypothalamic–Pituitary–Ovarian Axis

An intriguing group of autoantibodies includes those targeting the hypothalamic–pituitary–ovarian (HPO) axis, which have also been detected in women with PCOS. These include autoantibodies against gonadotropin-releasing hormone receptor (GnRHR) [67,68,69,70], follicle-stimulating hormone (FSH) [71], follicle-stimulating hormone receptor (FSHR) [72], luteinizing hormone receptor (LHR) [72], and ovarian tissue [73]. Given the critical role of the HPO axis in hormonal regulation in PCOS, these autoantibodies warrant separate consideration, as their interaction with these key regulatory organs may have a direct pathogenic role in the development of PCOS [74].

The findings regarding each of these autoantibodies are inconsistent; however, among them, most reports focus on the relevance of anti-FSH antibodies [71,75], antiovarian antibodies (AOAs) [73,76,77], and anti-GnRHR antibodies [68,69,78,79]. In the case of anti-FSH antibodies, available data suggest a potential neutralizing effect, as they have been shown to bind to the V14D epitope of the FSH β-chain, which is critical for proper receptor binding [71]. The exact role of AOAs is less well understood, as they represent a heterogeneous group of antibodies directed against various ovarian tissue antigens [73]. They may therefore be a marker of secondary ovarian damage resulting from PCOS, or they may also exert a neutralizing effect.

Anti-GnRHR antibodies appear particularly intriguing, as they have been demonstrated to target the second extracellular loop (ECL2) of the gonadotropin-releasing hormone receptor and act similarly to activating anti-TSHR antibodies in Graves’ disease [69,80]. Continuous, unopposed activation of GnRHR by these antibodies may disrupt the physiological pulsatile secretion of GnRH, which is essential for the normal function of the HPO axis [69]. This dysregulation is consistent with the elevated GnRH pulse frequency observed in PCOS, a prominent driver of LH excess and relative FSH deficiency [81]. Functionally, the pathogenic potential of activating anti-GnRHR antibodies has been confirmed in cell-based GnRHR assays through inhibition by the GnRHR antagonist cetrorelix, which was able to block receptor activation mediated by these autoantibodies [67,69]. Furthermore, an autoimmune PCOS rat model was developed by immunizing animals with a synthetic peptide derived from the GnRHR-ECL2 sequence [79]. Effective inhibition of GnRHR-AAb activity in a cell-based bioassay was achieved using an epitope-mimicking retro–inverso peptide inhibitor (d-CHTVCQSF) [78]. These findings strongly suggest that elevated anti-GnRHR antibodies may play a direct pathogenic role in PCOS, and further research is warranted to assess their potential as therapeutic targets.

## 3. PCOS as a Chronic Low-Grade Inflammatory Disease

**Figure 1:**
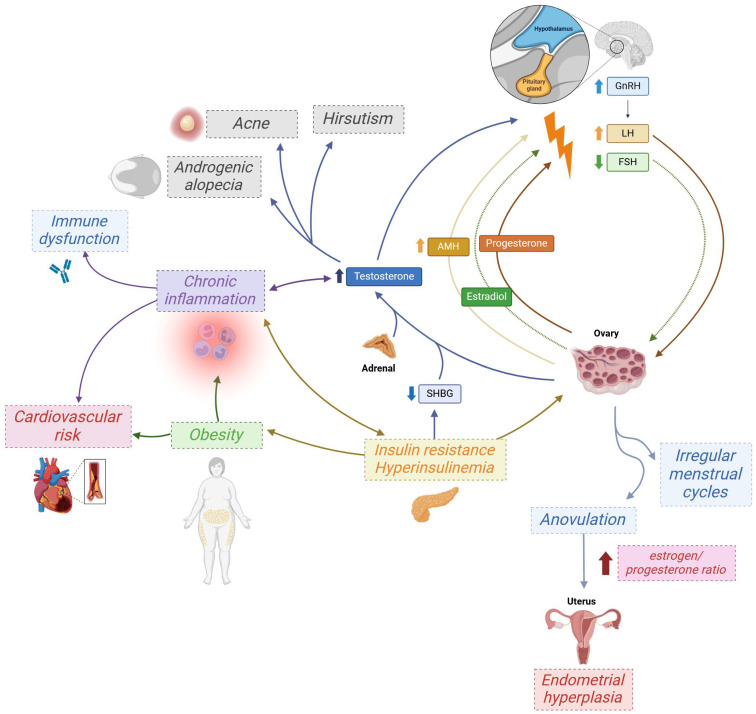
The pathophysiology of polycystic ovary syndrome (PCOS). Endocrine disorders in PCOS are a self-perpetuating process. Of particular note is the significant role of inflammation, which is closely associated with endocrine disruption and contributes to many of the complications of PCOS, including overstimulation of the immune system and consequent overproduction of autoantibodies. Upward and downward arrows indicate increased (↑) or decreased (↓) levels. GnRH—gonadotropin-releasing hormone; LH—luteinizing hormone; FSH—follicle-stimulating hormone; SHBG—sex hormone-binding globulin; AMH—anti-Müllerian hormone. Created with Biorender.com.

Polycystic ovary syndrome (PCOS) is unequivocally an inflammatory condition characterized by localized inflammation within the reproductive system and a low-grade, chronic systemic inflammatory process affecting the entire organism [82]. Up to 60% of women with PCOS are overweight or obese [83]. Not only is obesity itself an inflammatory condition, but the cascade of events it triggers exacerbates the pro-inflammatory state in the body of a woman with PCOS [84]. It is widely recognized that adipose tissue macrophages release tumor necrosis factor-α (TNF-α) and interleukin-6 (IL-6), which are involved in the development of insulin resistance [85]. Moreover, adipocytes themselves secrete pro-inflammatory factors like leptins, lipocalin, resistin, TNF-α, IL-6, and interleukin-1 (IL-1), entitled adipokines, further contributing to hyperinsulinemia, which exacerbates obesity and inflammation [86,87]. Additionally, obesity makes thecal cells more sensitive to luteinizing hormone (LH), ultimately leading to increased androgen production and hyperandrogenism, changes that may, through several metabolic and paracrine pathways, contribute to chronic low-grade inflammation in women with PCOS [88]. Elevated levels of pro-inflammatory markers are also commonly observed. Their increased concentration, caused by both PCOS and obesity, significantly influences the risk of developing other systemic diseases. A concise overview of the pathophysiology of PCOS is presented in Figure 1.

Physiologically, androgens exert predominantly immunomodulatory, anti-inflammatory effects, and androgen-receptor signaling is present in innate and adaptive immune cells [89,90]. In PCOS, however, hyperandrogenism coexists with insulin resistance and adiposity, making the directionality of associations difficult to disentangle [82]. Observational data linking circulating androgens and inflammatory markers are heterogeneous and often attenuate after adjustment for adiposity [82]. Interventional evidence is very limited. Combined oral contraceptives, including antiandrogenic progestins such as ethinylestradiol/cyproterone acetate and ethinylestradiol/drospirenone, are associated with higher C-reactive protein (CRP) in women with PCOS, as shown in a systematic review and meta-analysis [91]. This reflects an effect likely driven by hepatic actions of oral estrogens rather than androgen-receptor blockade per se [92]. The impact of antiandrogen monotherapy (e.g., spironolactone, flutamide/bicalutamide, finasteride/dutasteride) on inflammatory markers remains insufficiently studied. In a small study, bicalutamide did not change CRP in PCOS [93], and recent randomized trials of flutamide monotherapy did not include inflammatory markers as outcomes [94]. Likewise, there are no longitudinal data on autoantibody profiles with antiandrogen therapy and no PCOS-specific studies assessing inflammation or autoantibodies after oophorectomy, while the non-PCOS literature on bilateral oophorectomy indicates cytokine alterations, highlighting a knowledge gap [95].

**Figure 2:**
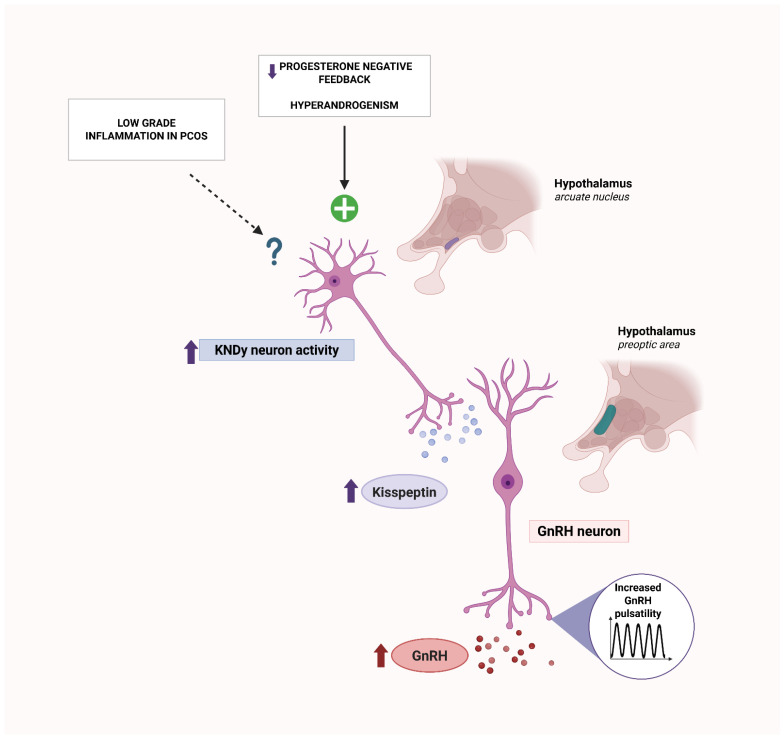
Kisspeptin in PCOS—steroid-feedback dysregulation and GnRH hyperpulsatility. Impaired progesterone negative feedback, together with hyperandrogenism, augments KNDy (arcuate) activity, increasing kisspeptin input to GnRH neurons and raising GnRH pulse frequency. The dashed arrow from low-grade inflammation indicates an uncertain/indirect central effect in PCOS. Solid arrows denote relationships supported by current evidence. Upward and downward arrows indicate increased (↑) or decreased (↓) activity or levels. GnRH—gonadotropin-releasing hormone; KNDy—kisspeptin/neurokinin B/dynorphin (neurons of the arcuate nucleus); PCOS—polycystic ovary syndrome. Created with Biorender.com.

Kisspeptin, a neuropeptide encoded by the *KISS1* gene and signaling via its G-protein-coupled receptor *KISS1R* (GPR54), is essential for puberty, fertility, and the upstream control of GnRH-releasing neurons, thereby coordinating HPO axis function [96]. In the arcuate nucleus, KNDy neurons (kisspeptin/neurokinin B/dynorphin) set GnRH/LH pulse frequency [96]; experimental attenuation of kisspeptin signaling slows LH pulsatility, establishing KNDy as the core GnRH/LH pulse generator [97]. Hormonal disturbances typical of PCOS, such as androgen excess together with impaired progesterone negative feedback at KNDy neurons, likely augment kisspeptinergic output, increasing GnRH pulsatility and LH secretion [98,99,100]. In PCOS, circulating kisspeptin is frequently elevated versus controls and often correlates with LH, which is consistent with increased GnRH pulse frequency in women with PCOS [101,102]. Interestingly, experimental studies in animal models indicate that inflammation can modulate kisspeptin signaling, thereby altering downstream GnRH/LH pulsatility. Acute exposure to lipopolysaccharide or interleukin-1β suppresses arcuate *KISS1* expression and blunts GnRH/LH pulsatility [103,104]. Chronic inflammatory models likewise reduce central kisspeptin tone [105]. These central inflammatory effects are inconsistent with the increased GnRH pulse frequency characteristic of PCOS and are therefore unlikely to account for this feature. By contrast, several clinical contexts with inflammation report higher serum kisspeptin [106,107], which likely reflects peripheral production rather than hypothalamic drive: *KISS1* and *KISS1R* are expressed in the ovary, adipose tissue, and the liver, where kisspeptin can be secreted as a glucagon-regulated hepatokine [106,108,109]. In an insulin-resistant PCOS phenotype, such peripheral sources may therefore contribute to elevated circulating kisspeptin, while central KNDy activity seems to remain primarily governed by steroid feedback [110,111]. Moreover, temporal coupling between endogenous kisspeptin and LH pulses is disrupted in PCOS, suggesting neuroendocrine network dyscoordination [112]; whether low-grade inflammation contributes to this dyscoordination remains to be determined. These relationships are summarized schematically in Figure 2.

Cytokines are small signaling proteins playing a crucial role in cell communication. They are produced by a variety of cells, including adipocytes and immune cells, but also oocytes or follicular cells [113,114]. These molecules, produced within the ovary, can act autocrinely to regulate ovarian metabolism by influencing processes such as ovarian cell proliferation, folliculogenesis, steroidogenesis, and hormonal balance [115]. Additionally, they can function paracrinely, affecting distant tissues and leading to systemic effects [116]. These findings are clinically significant for affected patients, as elevated inflammatory markers are associated with an increased risk of both systemic conditions, such as cardiovascular diseases, atherosclerosis, autoimmune disorders, and type 2 diabetes mellitus, and local complications within the reproductive system, including obstetric complications like miscarriages and placental insufficiency and those concerning follicular dysplasia [117,118,119]. Numerous studies have reported higher concentrations of inflammatory markers, including CRP, interleukin-18 (IL-18), tumor necrosis factor (TNF-α), interleukin-6 (IL-6), white blood cell count (WBC), ferritin, monocyte chemoattractant protein-1 (MCP-1), and macrophage inflammatory protein-1α (MIP-1α), as well as elevated concentrations of advanced glycation end products (AGEs) along with an upregulation of their receptor, receptor for advanced glycation end products (RAGE), in women with PCOS [117]. Interestingly, lower levels of anti-inflammatory cytokines, namely, omentin and adiponectin, have also been reported in obese patients with PCOS [117]. Some studies describe reduced levels of pentraxin-3 (PTX3), a protein structurally similar to CRP, which plays a regulatory role in the development of inflammation [120]. In addition to its regulatory function, PTX3 also exerts a protective effect against the development of atherosclerosis and cardiovascular diseases [121]. These findings suggest that the chronic inflammatory state in PCOS is not solely attributed to the elevation of pro-inflammatory markers, but also attributed to a reduction in anti-inflammatory mediators [82]. Some of these markers are particularly valuable in the clinical assessment of patients. For example, CRP, according to the American Heart Association, is a well-established prognostic marker for cardiovascular risk. Therefore, assessing whether CRP levels exceed 3 mg/L may be especially useful in patients with PCOS, as it can provide important insights into their cardiovascular risk profile [82].

An optimal immunological environment in the endometrium is essential for both the proper functioning of the protective barrier against pathogens and successful embryo implantation [122]. This already complex immunological landscape, typically rich in CD56+ uterine natural killer (uNK) cells, CD68+ macrophages, or CD8+ cytotoxic T lymphocytes, is distinctly altered in women with PCOS [123,124,125,126]. It appears that the endometrium of women with PCOS is characterized by a decreased density of uNK cells, reduced levels of anti-inflammatory factors such as haptoglobin and apolipoprotein A1, and elevated levels of pro-inflammatory markers including TNF-α, IL-6, and nuclear factor kappa B (NF-κB) [126]. Elevated TNF-α promotes the proliferation of endometrial cells and modifies estrogen metabolism, increasing the production of carcinogenic metabolites in the endometrium [127]. Furthermore, the increased inflammatory state alters insulin sensitivity, leading to the development of insulin resistance in the endometrium of women with PCOS. These intriguing findings are closely related to implantation and embryo tolerance, as they contribute to the creation of a suboptimal endometrial environment [126].

Most studies focus on searching for inflammatory markers in the plasma and endometrium rather than directly in the ovaries and follicles. However, there is growing evidence indicating that chronic inflammation is a key factor in the pathogenesis of follicular dysplasia [82]. Elevated levels of these pro-inflammatory markers lead to premature leukocyte infiltration, which may result in the destruction of follicular maturity [128]. Inflammation in this context is associated with abnormal follicular development, leading to mitochondrial dysfunction, which in turn causes impaired energy supply and ovarian insufficiency, affecting reproductive functions in women with PCOS [129]. A new and interesting direction in research is the possibility that this persistent excessive inflammation may lead to pyroptosis of granulosa cells, which is thought to contribute to anovulation and follicular atresia in PCOS [129].

At the transcriptomic level, inflammation is likewise evident in PCOS [130]. Network-based analyses in ovarian granulosa cells highlight MHC class II antigen processing and presentation (enrichment of *HLA-DQA1*, -*DQB1*, -*DRA*, -*DMA*, -*DPA1*), together with B-cell receptor signaling, Toll-like receptor, NF-κB pathways, phagosomes, Fc-γ-receptor signaling, and interferon response—a constellation consistent with conditions permissive for autoantibody generation [131]. In parallel, granulosa cell-focused RNA sequencing studies demonstrate enrichment of IL-6–JAK–STAT3, IL-2–STAT5, and PI3K–AKT–mTOR programs and identify high-inflammation molecular subtypes [132]. Whole-blood transcriptomics similarly reports dysregulation of inflammatory-response genes [133]. Recent multi-omics integrating single-cell and bulk transcriptomes also points to complement-cascade activity in PCOS ovaries [134]. These transcriptomic signatures are concordant with epigenetic findings reported earlier (EWASs of enrichment in MHC/HLA-linked and T1D-related pathways [51,52]).

PCOS is characterized as an inflammatory disease, with the inflammatory state not only affecting the reproductive organs but also influencing peripheral organs, impacting overall functioning of the body. The question remains whether this inflammation contributes to the development of an autoimmune response and the production of autoantibodies, whose elevated levels are observed in PCOS, emerging as a consequence of chronic inflammation, or whether these autoantibodies could be fundamental to the pathogenesis of PCOS itself.

## 4. Autoantibodies as Markers of Inflammation

Autoantibodies are now emerging as a marker for many autoimmune diseases with an inflammatory component, such as multiple sclerosis [135], lupus nephritis (LN) [136], and other rheumatic diseases [137]. The presence of autoantibodies has been correlated with either specific biomarkers or clinical criteria of the disease. Can the same principle be applied to PCOS? To answer this question, one should first consider whether a rise in autoantibody levels in PCOS is probable, and how this could fit into the pathology of the disease.

### 4.1. Mechanisms of Autoantibody Production: Insights from Rheumatoid Arthritis and Systemic Lupus Erythematosus

There are no studies on the mechanism of autoantibody production under inflammatory conditions in PCOS. However, in well-known rheumatic diseases, these processes are well understood. The molecular mechanisms behind autoantibody production under inflammatory conditions can be studied by looking at a model disease, rheumatoid arthritis (RA). There are two distinct molecular mechanisms by which autoantibodies arise under inflammatory conditions: by anti-citrullinated protein antibody (ACPA+) B cells or by rheumatoid factor (RF+) B cells [138]. Single-cell RNA sequencing revealed that ACPA+ and RF+ B cells are imprinted with distinct transcriptional programs. ACPA+ B cells develop within ectopic lymphoid structures (ELSs) in inflamed synovial tissue, where chronic antigen exposure, T cell help, and peptidyl arginine deiminase (PAD) enzyme-driven citrullination promote the production of high-affinity ACPAs. In contrast, RF+ B cells are activated in extrafollicular regions through innate immune signals (e.g., Toll-like receptor (TLR) activation) and Fc receptor signaling, leading to rapid expansion, class-switch recombination, and amplification of inflammation. Despite these differences, both ACPA+ and RF+ B cell programs ultimately promote antibody class-switching, survival, and activation, reinforcing chronic inflammation in RA. However, the fact that these autoantibodies correlate with disease severity but are not directly pathogenic is supported by the effectiveness of TNF-α inhibitors such as Adalimumab (HUMIRA) [139]. This monoclonal antibody targets TNF-α, a key driver of inflammation, without directly affecting antibody production. The success of Adalimumab in controlling disease progression suggests that autoantibodies, while important as biomarkers, may not play a direct role in the pathological process.

Another prominent autoimmune and inflammatory disease, systemic lupus erythematosus (SLE), is characterized by the presence of antinuclear antibodies (ANAs) in at least 70% of patients [140]. These antibodies target nuclear components such as DNA, RNA, or RNA-binding proteins. A potential mechanism linking inflammation to ANA formation has been depicted [141]. Nuclear antigens released from apoptotic cells may trigger autoimmunization, particularly when the clearance of cellular debris is inefficient [142]. Failure to effectively remove apoptotic cells allows nuclear antigens to persist in the extracellular space, increasing the likelihood of immune recognition and autoimmune activation. Inflammatory signals further enhance antigen presentation by antigen-presenting cells, leading to the activation of autoreactive B and T lymphocytes. This immune dysregulation contributes to the production of ANA and other autoantibodies, intensifying the autoimmune response and driving disease progression [141].

### 4.2. Increased B-Cell Activity in PCOS

While these model diseases provide valuable insights, their pathogenesis likely differs significantly from that of polycystic ovary syndrome (PCOS). To establish whether the models can be applied in this context, the literature was screened for evidence of similar upregulation in immune pathways. Increased frequency and activity of CD19+ B cells—immune cells capable of antibody production—were reported in the peripheral blood of PCOS patients [143]. In a follow-up study, the authors demonstrated that targeting CD19+ B cells with an anti-CD19 antibody alleviated the pathological phenotype in an induced mouse model. This was reflected in lower serum levels of TNF-α and immunoglobulin M (IgM), as well as reduced apoptosis of granulosa cells [144]. Although animal models may not fully replicate human disease, this finding suggests that autoreactive B cells in PCOS play a dual role—both in autoantibody production and in promoting low-grade chronic inflammation, which is a key feature of the disorder. These results highlight the potential involvement of immune dysregulation in PCOS pathogenesis.

Increased B-lymphocyte activity has been demonstrated to be associated with hormonal imbalance in PCOS. Studies often highlight relative hyperestrogenism as a key driver of excessive immune system activity in PCOS [23,145], defined as an increased concentration of estrogen relative to a low concentration of progesterone. Estrogens have been shown to be associated with a number of autoimmune diseases and to affect multiple populations of immune cells, both in innate and adaptive immunity [146]. Estrogens have been demonstrated to induce a number of cytokines, including an increase in interleukin-4 (IL-4) in T helper 2 (Th2) lymphocytes, IL-1 in monocytes, IL-6 in T-cells, and interferon-γ in T helper 1 (Th1) cells [145]. 17β-estradiol has been shown to enhance B lymphocyte autoreactivity through multiple mechanisms. It facilitates lenient negative selection, allowing the survival of autoreactive B cells [147]. Moreover, it promotes autoantibody production in splenic B cells and upregulates the expression of antiapoptotic genes like BCL2, as well as SHP2 and VCAM, which contribute to the persistence of autoreactive B cells [146,148]. Low concentrations of progesterone in women with PCOS do not oppose the effects of estrogen. Hyperandrogenism should demonstrate immunosuppressive effects and counteract these phenomena [145]. However, androgens’ impact is more complex—their elevated levels may disrupt the normal balance of immune cells and contribute to a state of chronic inflammation [149].

### 4.3. Oxidative Stress

Antibodies against α-crystallins, a class of chaperone-like heat shock proteins (HSPs), have been found to be highly expressed in women with PCOS and other endocrine disorders [19]. While these proteins are most abundant in the lens of the eye, they are also present in muscle, spleen, skeletal, and adipose tissue. Notably, the α-crystallin isoform has been detected in the sera of women experiencing spontaneous abortion, failed IVF, and unexplained infertility—further underscoring its relevance in the context of PCOS and endocrine dysfunction [19]. Additionally, α-crystallins have been identified in mouse ovaries, suggesting a potential role in ovarian physiology and pathology [150]. Moreover, the levels of anti-α-crystallin antibodies may rise in inflammatory states due to the unique properties of the αB-crystallin promoter. This promoter is highly responsive to cellular stress and can drive increased expression under conditions of prolonged oxidative stress [151]. Given that oxidative stress is a well-known contributor to PCOS pathology, either co-occurring with inflammation due to mitochondrial damage or directly inducing inflammatory responses, the involvement of α-crystallins in PCOS warrants further investigation.

It is highlighted that oxidative stress—closely tied to inflammation—may take part in triggering autoantibody production in PCOS [61]. Women with PCOS were found to have elevated levels of malondialdehyde-modified human serum albumin (HSA-MDA), a byproduct of oxidative stress and inflammation. Reactive oxygen species (ROS) can modify proteins and lipids, leading to the formation of new molecular structures, or “new epitopes”, that the immune system may recognize as foreign, potentially inducing autoantibody production. Additionally, increased levels of antibodies against HSA-MDA (anti-HSA-MDA) were detected in PCOS patients. Similar findings have been reported in other inflammatory conditions, including atherosclerosis, diabetes, essential hypertension, and renal failure [152,153,154]. These observations further support the idea that chronic inflammation in PCOS may contribute to the development of autoantibodies, reinforcing the role of immune dysregulation in the disease.

### 4.4. Release of Nuclear and Mitochondrial Antigens

Another hallmark of inflammation-driven autoimmune activity, as demonstrated in SLE [141], is the release of cellular content, including nuclear antigens, which can trigger autoimmunization. Evidence suggests that similar mechanisms may be relevant in PCOS; however, as discussed above, findings remain inconsistent [58]. One study found that serum levels of anti-dsDNA and anti-histone antibodies were significantly elevated in women with PCOS compared to controls, while ANA levels showed no significant difference between the groups [56], although another one showed a significantly higher prevalence of ANA positivity among women with PCOS compared to controls, suggesting a potential autoimmune component [22]. Similarly, Makled et al. reported an increased prevalence and elevated titers of ANAs and anti-dsDNA antibodies in PCOS patients compared to healthy individuals [63].

Beyond nuclear antigens, mitochondrial dysfunction has also been implicated in PCOS [149,155]. The observation that maternal inheritance plays a stronger role than paternal inheritance in the disease [5] further supports this hypothesis. Free mitochondrial DNA (mtDNA) can act as an autoantigen if it escapes from the mitochondria. The release of mtDNA can activate inflammatory pathways, including the NLRP3 inflammasome and interferon signaling, leading to immune activation [156].

**Figure 3:**
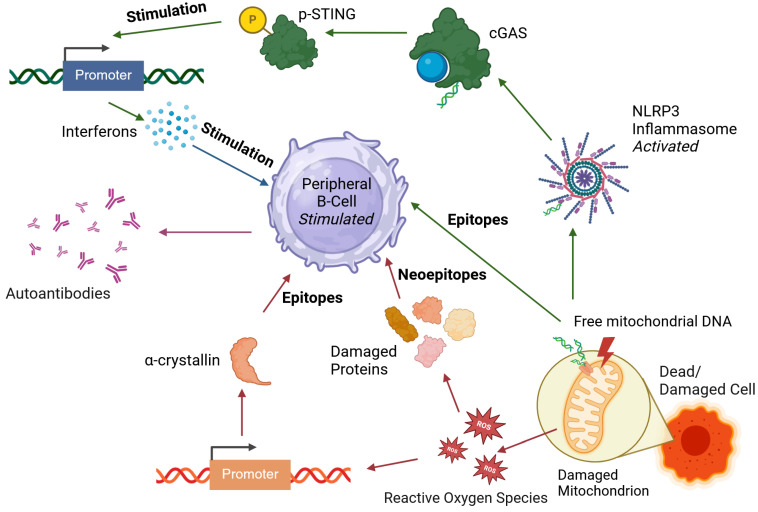
A proposed pathway of autoantibody generation in polycystic ovary syndrome (PCOS). The inflammation can arise from mitochondrial damage, which also triggers oxidative stress in the cell. The latter leads to increase in αB-crystallin promoter activity, generating epitope for autoimmunization, leading to production of autoantibody type found in PCOS. Furthermore, oxidative stress damages proteins, such as human serum albumin, leading to an increase in their modified version in the blood. These proteins then serve as neoepitopes. Mitochondrial damage leads not only to NLRP3 inflammasome assembly and activation, but also to release of oxidated fragments of mitochondrial DNA (mtDNA), which are another potential epitope for autoimmunization. The inflammasome then leads to activation of cyclic GMP-AMP synthase (cGAS), which triggers phosphorylation of stimulation of interferon genes (STING) protein. The process of self-reactive antibody generation is then mediated via activated peripheral B cells (also found in PCOS) in which, due to affinity maturation upregulation, the process has become more error-prone and self-reactive cells are not efficiently screened out. It is important to note that this is a proposed pathway based on a literature screening as described in this chapter. Created with Biorender.com.

Together with the increased inflammatory markers observed in PCOS [117], these findings suggest potential mechanisms for the emergence of autoantibodies in the disease, as summarized in Figure 3. The presented mechanisms and observations support the hypothesis that at least a portion of the autoantibodies detected in PCOS arise as a consequence of chronic low-grade inflammation.

## 5. Drivers of Elevated Autoantibody Levels in PCOS

**Figure 4:**
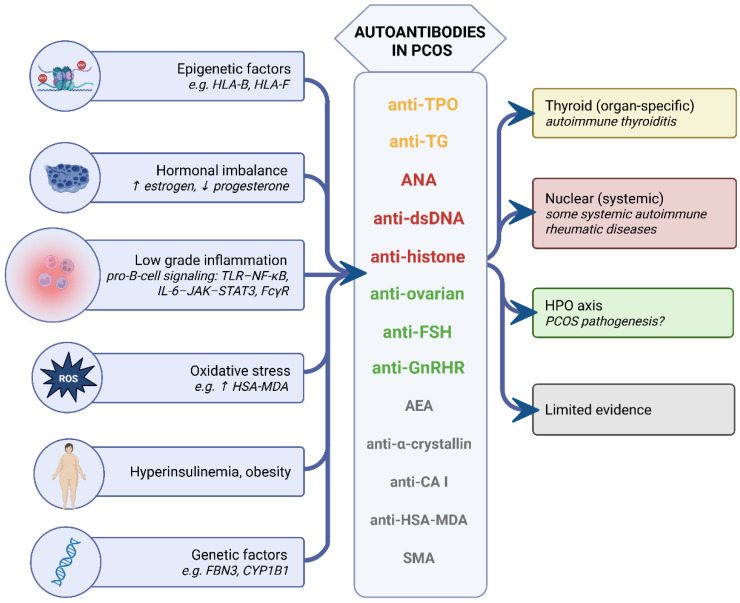
Elevated autoantibodies in PCOS—drivers and consequences. The left panel presents contributing factors that may promote autoantibody generation in PCOS. The center panel lists autoantibodies reported as elevated in PCOS; larger font denotes stronger evidence, whereas smaller text indicates limited evidence. The right panel shows consequence boxes color-matched to the corresponding autoantibodies. Upward and downward arrows indicate increased (↑) or decreased (↓) levels. AEAs—anti-endometrial antibodies; ANAs—antinuclear antibodies; anti-CA I—anti-carbonic anhydrase I antibodies; anti-dsDNA—anti-double-stranded DNA antibodies; anti-FSH—anti-follicle-stimulating hormone antibodies; anti-GnRHR—anti-gonadotropin-releasing hormone receptor antibodies; anti-HSA–MDA—antibodies to malondialdehyde-modified human serum albumin; anti-TG—anti-thyroglobulin antibodies; anti-TPO—anti-thyroid peroxidase antibodies; CYP1B1—cytochrome P450 family 1 subfamily B member 1; FBN3—fibrillin 3; FcγR—Fc gamma receptor; GnRH—gonadotropin-releasing hormone; GnRHR—gonadotropin-releasing hormone receptor; HLA-B/HLA-F—human leukocyte antigen B/F; HPO—hypothalamic–pituitary–ovarian; HSA–MDA—malondialdehyde-modified human serum albumin; IL-6—interleukin-6; JAK–STAT3—Janus kinase–signal transducer and activator of transcription 3 pathway; NF-κB—nuclear factor κB; PCOS—polycystic ovary syndrome; SMA—smooth muscle antibodies; TLR—Toll-like receptor. Created with Biorender.com.

PCOS is undoubtedly a disorder characterized by immune system dysfunction, accompanied by the increased prevalence of various autoantibodies. While numerous studies have demonstrated this phenomenon, there remains a paucity of knowledge regarding the mechanism of autoantibody emergence in women diagnosed with PCOS. Many studies indicate relative hyperestrogenism in PCOS as a factor associated with elevated immune system activity [15,23], without undertaking a comprehensive examination of the underlying mechanisms. Despite the scarcity of research in this area, it appears that a number of factors may be responsible for the observed increase in the detection of autoantibodies in PCOS (Figure 4).

Firstly, autoimmune diseases manifest more frequently in association with PCOS, which can be partially explained by their shared genetic susceptibility (e.g., FBN3, CYP1B1, and GnRHR have common polymorphisms in autoimmune thyroiditis and PCOS [33]), and epigenetic [49,50] or hormonal dysregulation occurring in individuals with autoimmunity (e.g., hyperinsulinism in women with type 1 diabetes treated with subcutaneous insulin injections [47,48]). Nevertheless, the pathogenesis of PCOS and autoimmune diseases through shared autoantibodies cannot be excluded, as indicated by the presence of anti-TPO antibodies in ovarian follicular fluid [35].

Secondly, a significant proportion of the antibodies detected in women with PCOS have been implicated in the process of inflammation, which constitutes a hallmark of the disease [117]. Low-grade chronic inflammation has been shown to be a cause of immune system dysfunction, which can lead to B-lymphocyte hyperreactivity observed in PCOS [143] and the production of a variety of autoantibodies. Autoantibodies directed against neoepitopes, formed as a result of changes in the structure of molecules under the influence of reactive oxygen species [61], or those directed against components of the cellular nucleus released from disintegrating cells [56], bring obvious connotations of inflammation.

The autoimmune background of PCOS and the autoantibodies attributed to its pathogenesis remain one of the most controversial theses. The autoantibodies directed against the HPO axis constitute the most promising lead, although a separate analysis is required for a full description and detailed discussion of their mechanisms. Nevertheless, recent findings, especially in the context of autoantibodies activating the receptor for gonadotropin-releasing hormone (GnRH), are intriguing and promising [67,69,78].

## 6. Conclusions

Converging molecular, immunologic, and functional data indicate three non-mutually exclusive drivers of autoantibody formation in PCOS.

- Shared susceptibility: Genetic, epigenetic, and transcriptomic data converge on MHC/HLA-centered pathways, suggesting a permissive antigen-presentation background for autoimmunity in a subset of women with PCOS. Clinically, this endotype may justify targeted screening for thyroid autoimmunity (anti-TPO, anti-TG), while non-thyroid autoantibodies are insufficiently studied and require prospective validation.
- Inflammation and oxidative stress: The chronic low-grade inflammatory environment provides autoantigen supply (neoepitopes, nuclear antigens) and pro-B-cell signaling (TLR–NF-κB, IL-6–JAK–STAT3, complement, FcγR), plausibly facilitating seroconversion, especially in insulin-resistant or obese phenotypes. Anti-inflammatory or metabolic interventions should be investigated for their capacity to lower specific autoantibody titers, and the contribution of androgens to inflammatory regulation in PCOS should be clarified in interventional studies.
- Strongest functional signal: Anti-GnRHR antibodies show agonist-like activity, are blocked by cetrorelix, and induce PCOS-like features in vivo; these remain the most compelling pathogenic autoantibodies in PCOS. Prospective, bioassay-stratified studies should test whether targeted therapies benefit women with demonstrable anti-GnRHR activity and define practical selection criteria for such treatment.

## Figures and Tables

**Table 1:** Autoimmune diseases associated with polycystic ovary syndrome (PCOS). Prevalence, risk, and association measures based on recent observational studies, systematic reviews, and meta-analyses.

Associated Autoimmune Disease	Study	Year	Type of Study	Co-Occurrence Frequency/Risk
Type 1 diabetes mellitus	Bayona et al. [18]	2022	systematic review and meta-analysis	The overall prevalence of PCOS among women with T1D was estimated at 26% (95% CI: 19–34%).
	Escobar-Morreale et al. [24]	2016	systematic review and meta-analysis	The prevalence of PCOS in women with type 1 diabetes was 24% (95% CI: 15–34).
Autoimmune thyroiditis	Hu et al. [25]	2022	systematic review and meta-analysis	The mean prevalence of HT in PCOS patients was 25.24%. PCOS patients had a higher risk of developing HT under a random-effects model (OR = 2.28, 95% Cl: 1.61–3.22).
	Du et al. [26]	2013	systematic review and meta-analysis	The prevalence of AIT in PCOS patients was higher than that in control groups, with the effect size calculated as OR = 4.81, 95% CI: 2.88–8.04.
	Bahreiny et al. [27]	2024	systematic review and meta-analysis	A considerable association was detected between PCOS and the presence of AIT (OR = 2.38, 95% CI: 1.63–3.49).
Graves’ disease	Chen et al. [28]	2020	retrospective cohort study	Women with GD could be at risk of developing PCOS. The adjusted HR of PCOS for patients with GD relative to patients without GD was 1.47, 95% CI: 1.09–1.98.
Rheumatoid arthritis	Merlino et al. [29]	2003	prospective cohort study	The development of RA in elderly women showed association with PCOS (RR = 2.58, 95% CI: 1.06–6.30).
Sjögren’s disease	McCoy et al. [30]	2022	retrospective case–control study	PCOS, including ovarian cysts and hirsutism, was associated with greater RR for SjD (RR = 1.65, 95% CI: 1.28–2.12).
Psoriasis	Lee et al. [31]	2020	retrospective population-based cohort study	The risk of psoriasis was higher in the PCOS group by an HR of 2.07 (95% CI: 1.25–3.43) compared with the control group.
Celiac disease	Nanah et al. [32]	2025	retrospective observational analysis	Women with celiac disease had higher odds of later women’s health conditions including PCOS (3.3% vs. 1.0%; OR = 3.2, 95% CI: 2.94–3.68).
Systemic sclerosis	Sharmeen et al. [16]	2021	retrospective study	Systemic sclerosis was significantly more frequent in the PCOS patients than the non-PCOS (0.40% vs. 0.0%, *p* = 0.0369).
Undifferentiated connective tissue disease	Sharmeen et al. [16]	2021	retrospective study	Undifferentiated connective tissue disease was significantly more frequent in the PCOS patients than the non-PCOS (0.53% vs. 0.0%, *p* = 0.0123).

PCOS—polycystic ovary syndrome; T1D—type 1 diabetes; CI—confidence interval; HT—Hashimoto thyroiditis; OR—odds ratio; AIT—autoimmune thyroiditis; GD—Graves’ disease; HR—hazard ratio; RA—rheumatoid arthritis; RR—relative risk; SjD—Sjögren’s disease.

**Table 2:** Overview of autoantibodies studied in women with polycystic ovary syndrome (PCOS).

Autoantibody	Study	Year	Results (PCOS vs. Controls)	Conclusions
anti-TPO	Arduc et al. [37]	2015	26.7% vs. 6.6% ***p* = 0.002** 2.8 (0.2–600) vs. 1.5 (0.2–95) IU/mL ***p* = 0.012**	A higher concentration of anti-TPO along with an increased occurrence of anti-TPO positivity was identified in women with PCOS in comparison to those in the non-PCOS control group. Interestingly, Pearson correlation analysis revealed a positive association between anti-TPO levels and estradiol, the estradiol/progesterone ratio, and TSH levels.
	Hepşen et al. [20]	2018	37.9% vs. 11.1% ***p* < 0.001** 52 (0.2–1300) vs. 10 (10–1000) IU/mL ***p* < 0.001**	Anti-TPO levels and anti-TPO positivity prevalence were significantly higher in euthyroid PCOS patients in comparison with controls.
	Janssen et al. [41]	2004	26.9% vs. 8.3% ***p* < 0.001** 123 ± 328 vs. 10 ± 18 IU/mL ***p* < 0.001**	Both anti-TPO levels and the prevalence of anti-TPO positivity were significantly elevated in PCOS patients compared to the control group. Although LH and FSH levels did not differ individually, the LH-to-FSH ratio was higher in antibody-positive patients.
	Kim et al. [42]	2019	4.8% vs. 7.6% *p* = 0.88	Neither anti-TPO positivity nor thyroid parenchymal changes suggestive of thyroiditis were more prevalent in women with PCOS than in controls.
	Adamska et al. [43]	2020	22.0% vs. 23.9% *p* = 0.07	The frequency of positive serum anti-TPO did not differ between women with PCOS and controls or among phenotypes A, B, and C. Interestingly, women presenting phenotype D were characterized by the lowest frequency of occurrence of positive anti-TPO.
anti-TG	Arduc et al. [37]	2015	16.2% vs. 5.0% ***p* = 0.039** 17.5 (0.9–1098) vs. 10.8 (0.9–239) IU/mL ***p* = 0.014**	The study demonstrated a higher prevalence of anti-TG levels in PCOS patients.
	Hepşen et al. [20]	2018	15.3% vs. 5.1% ***p* = 0.013** 26 (0.9–524) vs. 20 (10–308) IU/mL ***p* < 0.001**	Anti-TG antibody levels were determined to be significantly higher in the euthyroid PCOS group. Anti-TG antibody positivity prevalence of euthyroid PCOS patients was significantly higher as compared to controls too.
	Janssen et al. [41]	2004	26.9% vs. 8.3% ***p* < 0.001** 113 ± 312 vs. 4 ± 17 IU/mL ***p* < 0.001**	Anti-TG levels and the rate of anti-TG positivity were notably higher in PCOS patients compared to the control group. While LH and FSH levels were similar individually, the LH-to-FSH ratio was increased in patients with positive antibody results.
	Novais et al. [62]	2014	9.2% vs. 7.7% *p* = 0.7527	There was no difference between the two groups with respect to the presence of anti-TG antibodies.
ANAs	Rashid et al. [22]	2018	18.4% vs. 2.29% ***p* < 0.01**	Serum ANA positivity was significantly more prevalent in women with PCOS than in controls. It correlated with clinical signs of hyperandrogenism and plasma glucose but showed no significant link to other hormonal parameters.
	Makled et al. [63]	2015	36% vs. 6% ***p* < 0.001** 9.0 ± 6.1 vs. 5.4 ± 2.3 IU/mL ***p* < 0.001**	Mean serum ANA levels were significantly higher in women with PCOS than in controls and showed a notable association with TSH levels.
	Ibrahim et al. [64]	2019	8.0 ± 2.7 vs. 5.1 ± 2.6 IU/mL ***p* < 0.001**	Serum ANA levels were significantly higher in PCOS than in controls and were strongly correlated with FSH and LH.
	Petrikova et al. [65]	2015	0.66% vs. 2.7% *p* = 0.250	There were no significant differences in the prevalence of ANA between PCOS and controls.
anti-dsDNA	Hefler-Frischmuth et al. [56]	2010	4.6 ± 3.8 vs. 3.8 ± 1.6 IU/mL ***p* = 0.02**	Serum levels of anti-dsDNA were significantly higher in women with PCOS.
	Makled et al. [63]	2015	28% vs. 2% ***p* < 0.001** 56.3 ± 25.7 vs. 26.0 ± 10.8 IU/mL ***p* < 0.001**	Mean serum anti-dsDNA levels and prevalence of serum anti-dsDNA positivity were significantly higher in women with PCOS than in controls.
	Ibrahim et al. [64]	2019	54.2 ± 20.3 vs. 24.0 ± 15.0 IU/mL ***p* < 0.001**	Serum anti-dsDNA levels were significantly higher in PCOS compared to control women.
	Hamedi et al. [58]	2014	42.5 ± 38 vs. 35.4 ± 39 IU/mL *p* = 0.23	Serologic markers of autoimmunity, anti-dsDNA, were not elevated in PCOS patients.
anti-histone	Hefler-Frischmuth et al. [56]	2010	7.8 ± 7.7 vs. 5.5 ± 6.1 IU/mL ***p* = 0.02**	Serum levels of anti-histone antibodies were significantly higher in women with PCOS.
AEAs	Palacio et al. [61]	2006	mean AEA level (exact values not provided) ***p* < 0.01**	Women with PCOS had a significantly higher mean AEA level compared to the control group.
anti-α-crystallin	Buteva-Hristova et al. [19]	2017	25.4% vs. 0% ***p* = 0.029** 0.6031 (0.36–1.26) vs. 0.4979 (0.42–0.6) OD (492 nm) ***p* = 0.021**	In the PCOS group, the levels of anti-α-crystallin antibodies were significantly higher than in the control group. Moreover, the proportion of positive sera in this group was considerably greater compared to the control group.
anti-CA I	Menteşe et al. [66]	2013	26% vs. 5.3% ***p* < 0.05** 0.311 ± 0.180 vs. 0.190 ± 0.098 A (480 nm) ***p* < 0.0001**	Women with PCOS had significantly higher mean anti-CA I antibody levels, while anti-CA II levels showed no significant difference from controls. All patients positive for anti-CA II were also positive for anti-CA I.
anti-CA II			4% vs. 0% *p* > 0.05 0.332 ± 0.174 vs. 0.333 ± 0.107 A (480 nm) *p* > 0.05	
anti-HSA-MDA	Palacio et al. [61]	2006	0.09 ± 0.03 vs. 0.041 ± 0.03 A (620 nm) ***p* < 0.05**	Patients with PCOS had a significantly higher mean serum anti-HSA-MDA level compared to the control group.
SMAs	Reimand et al. [59]	2001	15% vs. 5.1% ***p* < 0.005**	SMAs were significantly more frequent in PCOS than in the control group.

Percentage values refer to antibody positivity prevalence. Numerical values are given as mean ± standard deviation or median (minimum–maximum), unless otherwise specified. Values are given in the order of PCOS vs. controls. Statistically significant results (*p* < 0.05) are highlighted in bold. Anti-TPO—anti-thyroid peroxidase; IU/mL—international units per milliliter; PCOS—polycystic ovary syndrome; TSH—thyroid-stimulating hormone; LH—luteinizing hormone; FSH—follicle-stimulating hormone; anti-TG—anti-thyroglobulin; ANAs—antinuclear antibodies; anti-dsDNA—anti-double-stranded DNA; AEAs—anti-endometrial antibodies; OD—odds ratio; anti-CA I—anti-carbonic anhydrase I; Anti-CA II—anti-carbonic anhydrase II; A—absorbance units; anti-HSA-MDA—anti-malondialdehyde-modified human serum albumin; SMAs—smooth muscle antibodies.

## Abbreviations

The following abbreviations are used in this manuscript:

A	absorbance units
ACPA+	anti-citrullinated protein antibody
AEAs	anti-endometrial antibodies
AGEs	advanced glycation end products
AIT	autoimmune thyroiditis
AMAs	antimitochondrial antibodies
AMH	anti-Müllerian hormone
ANAs	antinuclear antibodies
AOAs	antiovarian antibodies
anti-CA	anti-carbonic anhydrase antibodies
anti-dsDNA	anti-double-stranded DNA
anti-GAD65	anti-glutamic acid decarboxylase 65
anti-HSA-MDA	anti-malondialdehyde-modified human serum albumin
anti-IA2	anti-insulinoma-associated antigen-2
anti-SSA	anti-Sjögren’s-syndrome-related antigen A
anti-TG	anti-thyroglobulin
anti-TPO	anti-thyroid peroxidase
anti-β2GPI	anti-β2-glycoprotein I antibodies
ARAs	antireticulin antibodies
cGAS	cyclic GMP-AMP synthase
CI	confidence interval
CRP	C-reactive protein
ECL2	second extracellular loop
ELSs	ectopic lymphoid structures
EWASs	epigenome-wide association studies
FSH	follicle-stimulating hormone
FSHR	follicle-stimulating hormone receptor
GD	Graves’ disease
GDM	gestational diabetes mellitus
GnRH	gonadotropin-releasing hormone
GNRHR	gonadotropin-releasing hormone receptor
GWASs	genome-wide association studies
HLA	human leukocyte antigen
HPO	hypothalamic–pituitary–ovarian
HR	hazard ratio
HSP	heat shock protein
HT	Hashimoto thyroiditis
IA2	anti-insulinoma-associated antigen-2
IgM	immunoglobulin M
IL-1	interleukin-1
IL-18	interleukin-18
IL-4	interleukin-4
IL-6	interleukin-6
INSR	insulin receptor
IU/mL	international units per milliliter
IVF	in vitro fertilization
KEGG	Kyoto Encyclopedia of Genes and Genomes
KNDy	kisspeptin/neurokinin B/dynorphin
LH	luteinizing hormone
LHR	luteinizing hormone receptor
LKMAs	liver/kidney microsomal antibodies
LN	lupus nephritis
MCP-1	monocyte chemoattractant protein-1
MCTD	mixed connective tissue disease
MHC	major histocompatibility complex
MIP-1α	macrophage inflammatory protein-1α
mtDNA	mitochondrial DNA
NF-κB	nuclear factor kappa B
OD	odds ratio
OR	odds ratio
PAD	peptidyl arginine deiminase
PCAs	parietal cell antibodies
PCOM	polycystic ovarian morphology
PCOS	polycystic ovary syndrome
PTX3	pentraxin-3
RA	rheumatoid arthritis
RAGE	receptor for advanced glycation end products
RF+	rheumatoid factor
ROS	reactive oxygen species
RR	relative risk
SHPG	sex hormone-binding globulin
SjD	Sjögren’s disease
SLE	systemic lupus erythematosus
SMA	smooth muscle antibodies
STING	stimulation of interferon genes
T1D	type 1 diabetes
TGF-β	transforming growth factor β
Th1	T helper 1
Th2	T helper 2
TLR	Toll-like receptors
TMA	thyroid microsomal antibodies
TNF-α	tumor necrosis factor-α
Treg	regulatory T cell
TSH	thyroid-stimulating hormone
uNK	uterine natural killer
WBC	white blood cell count
